# Influence of Synthesis Conditions on the Capacitance Performance of Hydrothermally Prepared MnO_2_ for Carbon Xerogel-Based Solid-State Supercapacitors

**DOI:** 10.3390/gels11010068

**Published:** 2025-01-15

**Authors:** Vania Ilcheva, Victor Boev, Mariela Dimitrova, Borislava Mladenova, Daniela Karashanova, Elefteria Lefterova, Natalia Rey-Raap, Ana Arenillas, Antonia Stoyanova

**Affiliations:** 1 Institute of Electrochemistry and Energy Systems, Bulgarian Academy of Sciences, Acad. G. Bonchev Str., bl. 10, 1113 Sofia, Bulgaria; v.boev@iees.bas.bg (V.B.); mariela.dimitrova@iees.bas.bg (M.D.); borislava.mladenova@iees.bas.bg (B.M.); edl@iees.bas.bg (E.L.); 2 Institute of Optical Materials and Technologies, Bulgarian Academy of Sciences, Acad. G. Bonchev Str. bl.109, 1113 Sofia, Bulgaria; dkarashanova@yahoo.com; 3 Instituto de Ciencia y Tecnología del Carbono, INCAR-CSIC, Francisco Pintado Fe 26, 33011 Oviedo, Spain; nataliarey@uniovi.es (N.R.-R.); aapuente@incar.csic.es (A.A.)

**Keywords:** MnO_2_, carbon xerogel, hydrothermal synthesis, solid-state supercapacitors

## Abstract

In this study, the potential to modify the phase structure and morphology of manganese dioxide synthesized via the hydrothermal route was explored. A series of samples were prepared at different synthesis temperatures (100, 120, 140, and 160 °C) using KMnO_4_ and MnSO_4_·H_2_O as precursors. The phase composition and morphology of the materials were analyzed using various physicochemical methods. The results showed that, at the lowest synthesis temperature (100 °C), an intercalation compound with composition K_1.39_Mn_3_O_6_ and a very small amount of α-MnO_2_ was formed. At higher temperatures (120–160 °C), the amount of α-MnO_2_ increased, indicating the formation of two clearly distinguished crystal structures. The sample obtained at 160 °C exhibited the highest specific surface area (approximately 157 m^2^/g). These two-phase (α-MnO_2_/K_1.39_Mn_3_O_6_) materials, synthesized at the lowest and highest temperatures, respectively, and containing an appropriate amount of carbon xerogel, were tested as active mass for positive electrodes in a solid-state supercapacitor, using a Na_+_-form Aquivion^®^ membrane as the polymer electrolyte. The electrochemical evaluation showed that the composite with the higher specific surface area, containing 75% manganese dioxide, demonstrated improved characteristics, including 96% capacitance retention after 5000 charge/discharge cycles and high energy efficiency (approximately 99%). These properties highlight its potential for application in solid-state supercapacitors.

## 1. Introduction

Recently, the growing concern for environmental protection on a global scale and the pursuit of a sustainable “green” economy provoked in-depth research into new materials and the development of advanced technologies for creating effective energy storage devices such as a new generation of batteries and supercapacitors. Among various energy storage devices, supercapacitors (SCs) stand out due to their unique properties, e.g., high power density, fast charge/discharge times, and long-term cycling stability [1], offering a number of possibilities for specific demands in many modern consumer and industrial applications, e.g., mobile devices, consumer electronics, and electric vehicles [2,3]. Solid-state supercapacitors with polymer electrolytes represent the next generation of energy storage devices, offering significant advantages over traditional liquid systems, particularly in safety by eliminating leakage and corrosion risks. Their compact and lightweight designs are especially beneficial for portable and wearable electronics, medical devices, smart textiles, and more [4].

The efficiency of these systems is highly dependent on the choice of electrode materials, which play a significant role in achieving adequate values of their capacity and electrochemical performance. In this context, manganese dioxide (MnO_2_) was a subject of increased research interest due to its remarkable properties such as high theoretical specific capacity [5,6] and a wide electrochemical potential window of approximately 0.9–1.0 V [1,7]. Despite the high value of the theoretical specific capacitance (1370 F/g), a main limiting issue is the insufficient utilization of the active mass of MnO_2_ electrodes. This is due to their poor electronic conductivity (10^−5^–10^−6^ S/cm), low ionic diffusion constant (10^−13^ cm^2^/Vs), and low structural stability [6,8], which cause a limitation to the bulk pseudo-capacitive reaction and leads to a significant reduction in the specific capacitance [9] and to poor electrochemical performance of the supercapacitor cells with this type of electrodes.

To address these drawbacks, considerable research work was conducted over the years to improve the electrochemical response of MnO_2_ by combining it with highly conductive carbon materials such as carbon nanotubes [10,11,12], graphene [13,14,15], carbon nanofibers (CNF) [16,17], N-doped carbon [18], conducting polymers [19,20], noble [21,22] and transition metals [23,24], etc., in order to exploit the synergistic contributions from all individual components by enhancing the specific parameters such as conductivity, specific capacitance, structural flexibility, and mechanical stability [19].

Moreover, since the electrochemical behavior of MnO_2_-based electrodes is strongly influenced by their morphological characteristics (e.g., dimensionality, crystal size, and porosity), the type of crystal modifications, structural defects, the specific surface area of the electrode, and the type of electrolyte used [8], numerous studies were carried out to elucidate the influence of MnO_2_ crystallinity on the electrochemical properties of SCs by examining their capacity values. It was shown that a key role for the supercapacitor’s applicability of such electrodes is played by the structural modification of manganese dioxide, which is known to exists in various polymorphs, each exhibiting unique structural and electrochemical properties [25]. Among these, the α-MnO_2_ phase, characterized by a 1D tunnels ((2 × 2) and (1 × 1)) in the tetragonal unit cell [26], was identified as particularly favorable for supercapacitor applications. This is due to its ability to store charge through ion intercalation, which is made possible by the sufficiently large tunnel size (4.6 Å), allowing the transfer of most of the alkali and alkaline earth metal cations (e.g., K^+^, Li^+^, Na^+^, Mg^2+^, Ca^2+^, etc.). Another modification suitable for supercapacitor electrode materials is δ-MnO_2_, which has a 2D layered structure with a layer spacing of about 7 Å, allowing the transport of water molecules and metal cations [27].

The preparation of the different polymorphs of MnO_2_ can be carried out by various widespread synthesis techniques, such as chemical co-precipitation [28], sol–gel [29,30], hydrothermal synthesis [31], thermal decomposition [32,33], electrochemical methods [34], etc., which are capable of providing the desired defined type of structure [35]. A very promising and currently widely used synthesis technique for the preparation of manganese oxide as an active material for supercapacitors is the hydrothermal method, which makes possible a variety of nanoarchitectures of MnO_2_ particles (such as flowerlike nanostuctures, α-MnO_2_ nanorods, nanotubes, nanofibers, etc.) to be prepared by changing the hydrothermal synthesis conditions—time, pressure and temperature [31,36]. It is evident that this method allows for the optimization of the synthesis conditions, leading to the production of materials with optimal properties, including improved ionic transport and increased active surface area. X. Bai et al. [31] proved in their research on MnO_2_-based electrodes the possibility of obtaining a hierarchical multidimensional MnO_2_ architecture built from particles with mixed morphology—cauliflower-like δ-MnO_2_ and α-MnO_2_ nanorods. The active material obtained in this way demonstrated promising results in terms of high specific capacity compared to single-phase MnO_2_ electrode materials. In addition to the benefits of hydrothermally prepared MnO_2_, research showed that potassium ion intercalation into the structure of layered MnO_2_ (Birnessite) significantly increases the specific surface area, improving the contact between the electrolyte and the electrodes and enhancing diffusion kinetics. In this regard, Li et al. [37] reported a superior rate performance of K-Birnesitte-based electrode materials for potassium-ion batteries, while Qu et al. [38] established good performance in asymmetric supercapacitors based on activated carbon/K-Birnessite as the active material.

Building on this approach, we hypothesized that combining α-MnO_2_ and potassium intercalate could further improve the electrochemical performance of the material facilitating ion transport and access to the active sites in the supercapacitor electrode material. The aim of this work was to study the physicochemical properties of the combined α-MnO_2_/K-Birnessite phase and correlate these properties with the electrochemical performance and stability of a composite electrode based on this two-phase material mixed with an activated carbon xerogel, which is a well-known synthetic porous material, combining a highly porous structure with high electrical conductivity [39]. We supposed that such a combination (i.e., carbon/manganese oxide) could exhibit a synergetic effect, leading to significant improvement in the electrochemical performance of supercapacitors.

## 2. Results and Discussion

### 2.1. Physicochemical Characterization

#### 2.1.1. XRD

**Figure 1:**
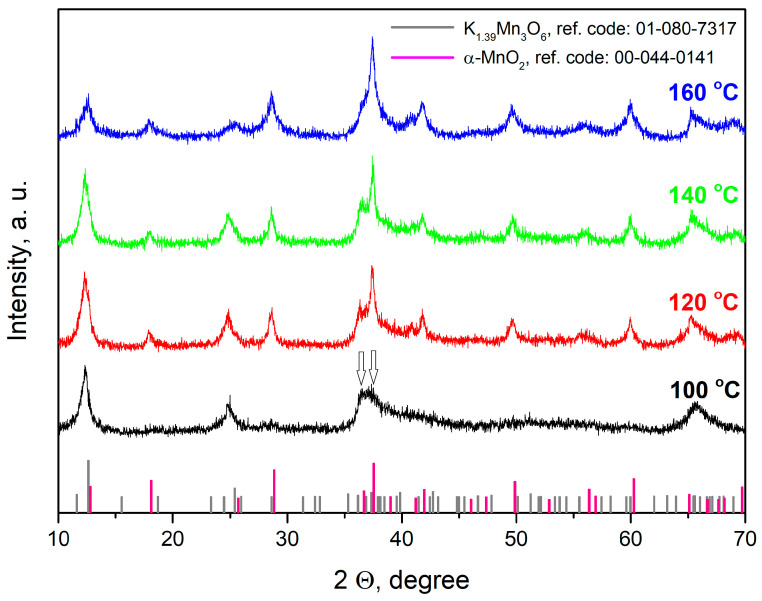
XRD patterns of samples hydrothermally synthesized under the present experimental conditions (VV0 at 100 °C, VV1 at 120 °C, VV2 at 140 °C, and VV3 at 160 °C) for 2 h.

The XRD patterns of the manganese oxide nanomaterials synthesized at different temperatures (100–160 °C) are presented in Figure 1. The analysis showed that, at the lowest temperature (100 °C), one main phase was formed with the most intensive peak at 12.62° 2θ and others at 25.39°, 37.33°, and 65.6° 2θ, corresponding to (−201), (402), (−112), and (021) crystallographic planes of the K_1.39_Mn_3_O_6_ structure, identified as a monoclinic phase (PDF-2 # 01-080-7317). The barely perceptible peak separation, registered at approximately 37° 2θ (marked with two arrows in the figure), along with a very weak peak around 29° 2θ, can be attributed to the presence of a small amount of the α-MnO_2_ phase, which forms during the initial stage of crystallization. The poorly resolved peaks of this phase overlap with those of the main phase, resulting in an apparent single peak due to the close proximity of their standard reflections and the relatively low content of the α-MnO_2_ phase. At a temperature of 120 °C, the peaks corresponding to this second phase (α-MnO_2_) become increasingly pronounced. The high-intensity peak at 37.52° 2θ, along with additional peaks at 18.11°, 28.84°, and 60.27°, correspond to the (211), (200), (310), and (521) planes of the tetragonal α-MnO_2_ structure (PDF-2 #00-044-0141), indicating a more prominent formation of this phase. A clear trend of change in peak intensity is observed, which is an indication that the amount of α-MnO_2_ increases with increasing processing temperature.

The crystallite sizes (d) were determined using Scherer’s formula: d = κλ/Bcosθ, where k is the shape factor, typically close to 1, λ (Å) is the wavelength, θ is the diffraction angle of the peak, and B (rad) is the line broadening at the full width at half maximum (FWHM) values of the peaks. The size of the crystallites in the main directions of the two phases formed varies from 4 to 16 nm (see Table 1).

The unit cell parameters and the amounts of the two identified phases in the obtained materials were calculated by Rietveld refinement using the MAUD software (version 2.9993), with the obtained results also presented in Table 1. The amount of α-MnO_2_, determined by this analysis, is difficult to detect in the XRD pattern of the sample prepared at 100 °C (α-MnO_2_—1.52%) due to its low content. As the synthesis temperature increases, the amount of α-MnO_2_ also increases, reaching 70.8% at 160 °C.

A change in the manganese oxide unit cell parameters was observed following the initial temperature increase to 120 °C. During the subsequent stepwise increase to the maximum temperature of 160 °C, at which the α-MnO_2_ content reached 70%, only minimal changes in the unit cell parameters were noted. More pronounced structural changes were observed for K_1.39_Mn_3_O_6_, where the unit cell volume decreases with increasing temperature.

Generally, the results of the XRD analysis clearly prove the formation of a mixture of α-MnO_2_ and K_1.39_Mn_3_O_6_ with a different ratio between the two phases depending on the hydrothermal temperature.

#### 2.1.2. SEM and TEM

**Figure 2:**
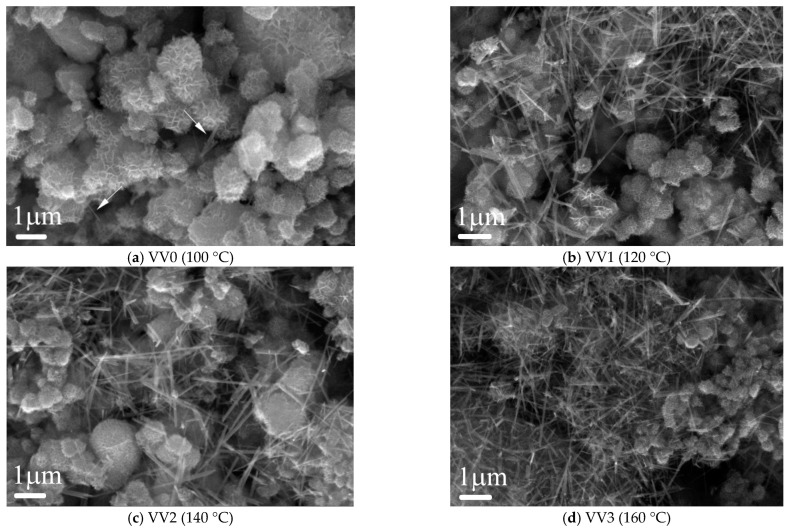
SEM images of hydrothermally synthesized samples at varying temperatures for 2 h. The arrows in (**a**) indicate a few isolated nanorods, manifesting the initial formation of α-MnO_2_ at 100 °C.

**Figure 3:**
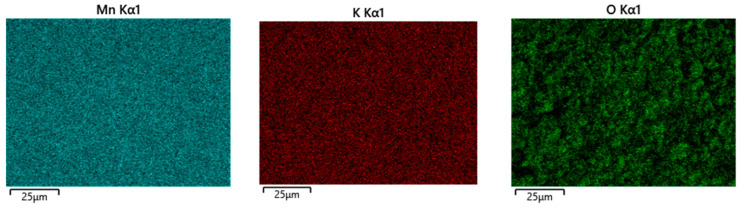
Element (Mn, K, and O) mapping images of K_1.39_Mn_3_O_6_· and α-MnO_2_ mixture in the VV3 sample.

The SEM analysis of the VV0–VV3 samples, prepared at different temperature conditions (Figure 2), clearly visualizes a noticeable morphology transformation with the increase in the hydrothermal temperature from 100 °C to 160 °C. It is evident that, at the lowest synthesis temperature (100 °C), the material demonstrates a cauliflower-like morphology, which is typical for the birnessite structural modification of MnO_2_, also identified by the XRD results as a K_1.39_Mn_3_O_6_ phase. Moreover, the image reveals a few isolated nanorods, manifesting the initial formation of α-MnO_2_ at this temperature (marked with arrows in Figure 2a). Increasing the temperature to 120 °C leads to a much more pronounced formation of nanorods, the morphology of which is typical of the α-MnO_2_ polymorph obtained by hydrothermal synthesis. (Figure 2b) [27]. The cauliflower-like structure remains mainly dominant up to 140 °C (Figure 2c), and a further increase in temperature to 160 °C causes the proportion of the nanorod-like contribution to predominate, as shown in Figure 2d. Additionally, the EDS elemental mapping images of the VV3 sample indicate a homogeneous distribution of Mn, K, and O in this sample (see Figure 3).

**Figure 4:**
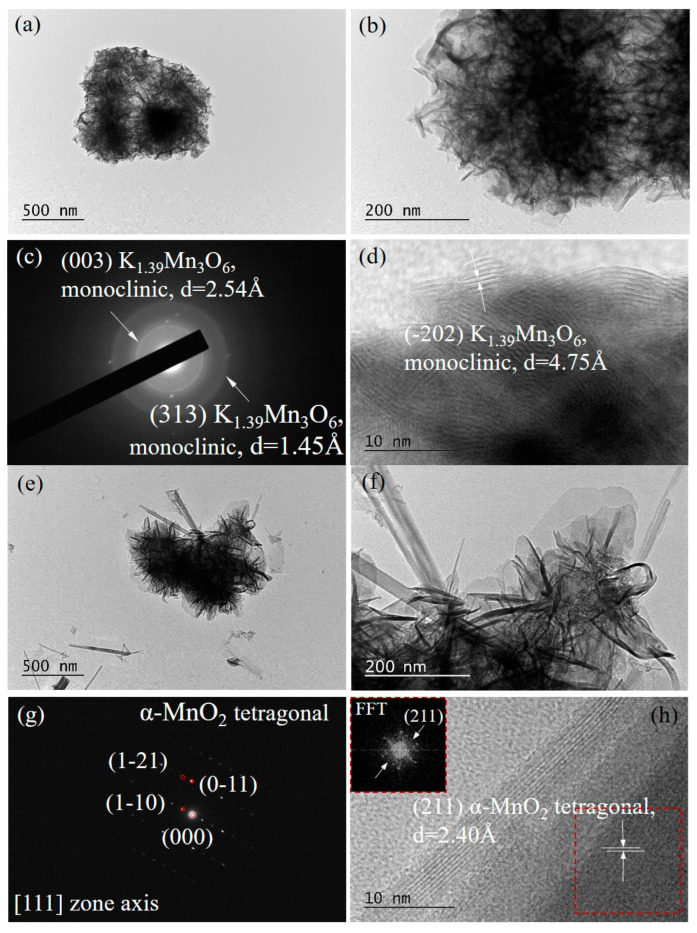
BF TEM micrographs at magnifications 10,000× (**a**,**e**) and 40,000× (**b**,**f**) and SAED pattern (**c**,**g**) for the VV0 and the VV3 samples, respectively, in each case. HRTEM for the VV0 sample (**d**) and HRTEM with FFT (**h**) and for the VV3 sample of the marked in red area as inset.

For a more detailed morphology characterization of the obtained MnO_2_-based materials, TEM analysis was performed. The comparison between the TEM micrographs of VV0 and VV3 samples in the BF TEM mode (Figure 4) reveals a clear morphological evolution corresponding to the temperature-dependent K-birnessite to α-MnO_2_ phase transformation, as already confirmed by SEM and XRD. Figure 4a,b show the cauliflower-like morphology consistent with the K-birnessite (K_1.39_Mn_3_O_6_) identified as a primary phase (98.48 wt% from the XRD results) in the low-temperature sample VV0. The indexing of the SAED pattern also proves the presence of the K-birnessite phase (Figure 4c) with an interplanar distance of 4.75 Å (Figure 4d), characteristic for (−202) family crystallographic planes of K_1.39_Mn_3_O_6_. The observed tightly packed structure predicts a lower porosity and specific surface area, which are confirmed below.

The TEM micrographs of the VV3 sample synthesized at 160 °C (Figure 4e,f) clearly visualize the dual-phase morphology with the coexistence of cauliflower-like and rod-shaped features. The nanorods are obviously dominating, which is indicative of a well-defined α-MnO_2_ phase [27].

#### 2.1.3. Porous and Surface Area Characterization

**Figure 5:**
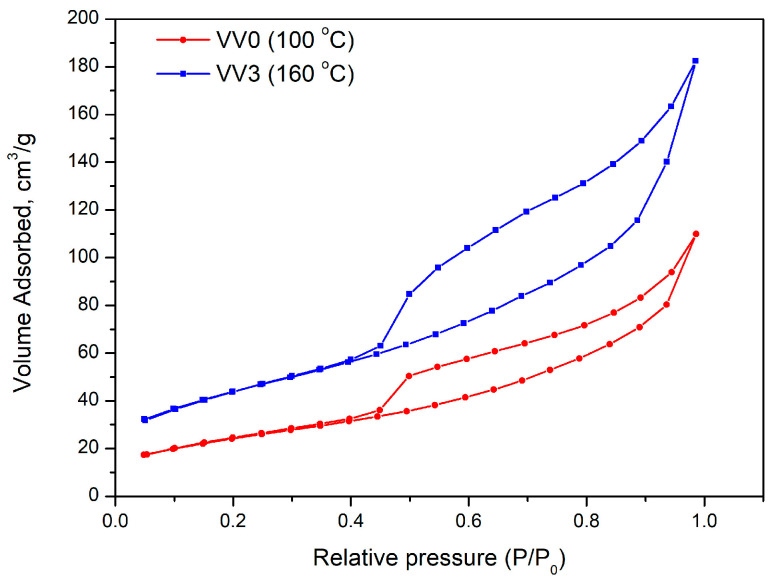
N_2_ adsorption–desorption isotherms of the samples, synthesized at 100 and 160 °C.

N_2_ adsorption–desorption isotherms for the VV0 and VV3 samples are shown in Figure 5. Both are type II according to the IUPAC classification, typical from an adsorption with no restrictions in macroporous solids.

However, the isotherm of the VV3 sample shows a higher N_2_ adsorption at low relative pressures than VV0, indicating a higher micropore volume and, therefore, a higher BET surface area (see Table 2). The increase in the volume adsorbed at medium and high relative pressures also clearly shows the presence of meso- and macroporosity and an influence of the synthesis temperature on the porous structure of the samples. The higher the temperature of synthesis, the higher the pore volume developed. The hysteresis loop (type H3 according to the IUPAC classification) is characteristic of materials having flaky morphology, with pores opening at both ends. However, it is also observed that the hysteresis loop starts and ends at the same relative pressures for both samples. This indicates that the mean size of the mesopores is analogous, as can be inferred from Table 2 (all samples have a mean pore size of ca. 4 nm). However, the size of the loop is different due to an increasing volume of mesopores developed as the synthesis temperature increases [40].

A clear trend in increasing porous properties (i.e., microporosity, BET surface area, mesopore volume, and total pore volume) with increasing synthesis temperature is observed. This phenomenon is likely mainly due to the formation of different polymorphic phases of the material, each of which is characterized by a different microstructure, as the temperature increases. At higher temperatures, certain phases (already identified by XRD analysis) exhibiting nanorods structures present higher porous properties resulting in a higher availability of active sites and less diffusional restriction.

### 2.2. Electrochemical Tests of the Assembled Cell

According to the physicochemical characterization results for the MnO_2_-based materials obtained and previous experience within the field, the dual-phase sample (α-MnO_2_/K_1.39_Mn_3_O_6_) with the highest specific surface area (VV3, 157 m^2^/g) was selected for electrochemical evaluation as a potential electrode material in hybrid supercapacitors. Additionally, the material synthesized at the lowest temperature (100 °C, VV0) was also tested for supercapacitor suitability due to the different phase composition (Table 1). Previous studies suggested that α-MnO_2_ enhances the electrochemical stability [27,41,42], while the potassium intercalation reduces the internal resistance [38], improving supercapacitor performance. A symmetric carbon xerogel-based supercapacitor was also used for comparison.

Optimizing the composition of the composite electrodes is critical to achieve the maximum synergy in the active electrode mass. The addition of activated carbon to MnO_2_-based materials improves the electrochemical properties by facilitating ion access, along with stabilizing the structure, which extends the electrode lifetime and improves the cycle stability compared with the material, where the birnessite phase predominates. Furthermore, the incorporation of carbon nanomaterials also enhances electrical conductivity, which also improves the electrochemical behavior of the electrode [43]. Therefore, two electrode compositions, 75 and 40 wt% MnO_2_-based materials, were selected in this study. The higher Mn oxide content (75%) is expected to enhance the energy storage capacity of the electrode, while the lower content (40%) is expected to improve the conductivity and stability due to the presence of the carbon matrix. This comparison can help to evaluate the optimal balance between performance, stability, and conductivity—key parameters for hybrid supercapacitor applications—and to identify which composition is most effective for practical use.

**Figure 6:**
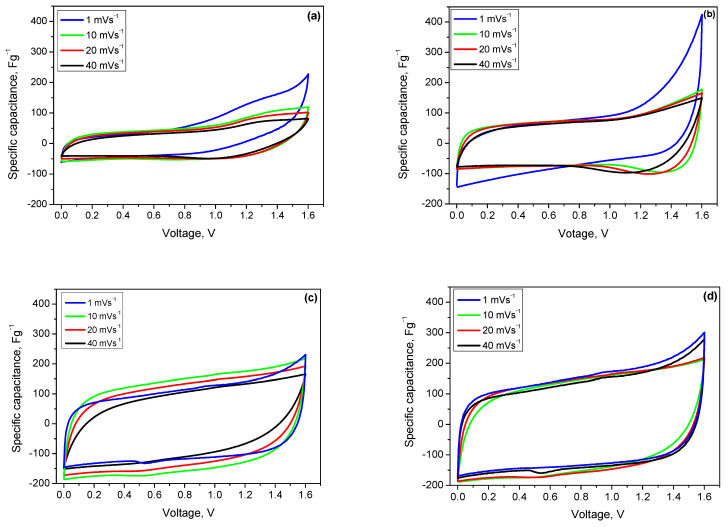
Cyclic voltammograms of solid-state asymmetric supercapacitors at scan rates of 1 to 40 mV/s, within a voltage window of 0.0 to 1.6 V and different composite electrodes: (**a**) VV0-75%; (**b**) VV0-40%; (**c**) VV3-75%; and (**d**) VV3-40%.

The cyclic voltammograms with the VV0 and VV3 samples, with the lowest and highest α-MnO_2_ contents, respectively, are shown in Figure 6. The voltammograms of VV3 display a typical profile for supercapacitor systems, with an almost rectangular shape. The curves overlap as the scan rate increases from 1 to 40 mV/s, indicating the excellent capacitive behavior of these devices [44]. For the VV0 sample, the rectangular shape of the CV profiles is less defined, and the curve at 1 mV/s differs significantly from those at higher scan rates, indicating the presence of secondary reactions that could affect the stability of the electrode.

The supercapacitor with VV0-based electrode demonstrates poorer performance compared to VV3, considering its lower surface area due to the negligible amount of α-MnO_2_ nanorods. The reason could be the limited charge storage capacitance caused by limited ion diffusion [45]. The presence of the activated carbon xerogel in the electrode seems to improve the capacitance but to a very low extent.

The comparison of the VV3 based electrodes with different Mn oxide contents (40% and 75%) shows much better electrochemical behavior, with higher capacitance, although, again, the presence of activated carbon xerogel in the electrode (Figure 6d) demonstrates negligible improvement. This could be explained by the key role of the Mn species in the charge storage mechanism, along with an optimization of the electrode composition (i.e., conductive additive amount), which provides a high amount of active material in the combination of effective electrical conductivity and stability of the electrode [46].

**Figure 7:**
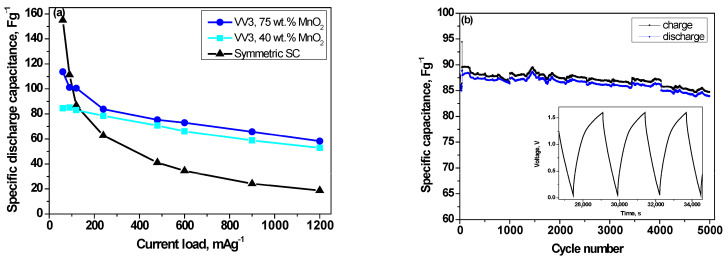
Discharge capacitance as a function of current load for symmetric and VV3 SCs (**a**) and cycling stability of discharge capacitance for 75% VV3 supercapacitors, with inset showing its voltage profiles (**b**).

In order to corroborate the results obtained, galvanostatic charge/discharge tests at different current loads and long-term cycling tests were performed on the supercapacitor containing the VV3 sample. To better understand them, Figure 7a shows the specific discharge capacitance for the VV3 sample and for a symmetric supercapacitor using activated carbon xerogel in both electrodes. The horizontal coordinate represents the applied current, which is identical for both the charging and discharging processes. The key role of the Mn oxide material in the charge storage is particularly evident, especially for the electrode containing 75% VV3, which shows stable capacitance with increasing current load. Its voltage profile, shown in Figure 7b (inset), deviates from the triangular shape, indicating the presence of Faradaic reactions and a resulting pseudocapacitive effect [47]. These surface-controlled redox processes significantly enhance the system’s capacitance. Despite the Faradaic reactions, the minimal difference between the charge and discharge curves highlights low polarization losses, further corroborated by the symmetrical cyclic voltammetric curves of VV3-75% in Figure 6c, even at high scan rates.

Furthermore, long-term testing with up to 5000 charge/discharge cycles of the sample with 75% VV3 (Figure 7b) confirms the stable operation of the supercapacitor, with 96% capacitance retention, which is quite promising compared to the studies by other researchers who have found a capacitance retention of 90% after 3000 cycles in a three-electrode cell system, based on a MnO_2_ nanotube-based electrode under comparable current loading [9]. This result highlights the crucial role of the morphology and structure of the synthesized MnO_2_.

The voltage profile shown in the inset (Figure 7b) further confirms the hybrid nature of the developed supercapacitor, consistent with our assumptions about the Faradaic reactions occurring within it.

## 3. Conclusions

This study successfully developed an optimized active material for asymmetric supercapacitors applications by integrating a dual-phase mixture of α-MnO_2_/K_1.39_Mn_3_O_6_ with activated carbon xerogel. The hydrothermal synthesis temperature was identified as a key parameter for achieving the desired nanostructures. At 100 °C, K_1__.__39_Mn_3_O_6_ was predominantly formed, while, at 120–160 °C, a material with a higher amount of α-MnO_2_ appeared, which contributed to the formation of nanorods.

The material synthesized at 160 °C exhibits the highest specific surface area (approximately 157 m^2^/g) and increased porosity, which significantly improves its electrochemical performance. Furthermore, the optimization of the electrode composition is essential in improving cycling capacity and durability. The best results were achieved with a composite containing 75% manganese dioxide, which demonstrated 96% capacitance retention after 5000 charge/discharge cycles and a high energy efficiency of 99%.

These findings imply the importance of the controlled synthesis conditions and the precise optimization of the MnO_2_–xerogel ratio in achieving appropriate material performance. This approach provides a strong foundation for the future development of high-performance materials tailored for long-term solid-state supercapacitor applications.

## 4. Materials and Methods

### 4.1. Synthesis Procedure

A series of four MnO_2_-based samples were prepared using the hydrothermal method, as reported in [31,48], varying the synthesis temperature. The synthesis was performed from initial components KMnO_4_ (Valerus Co., Sofia, Bulgaria) and MnSO_4_.H_2_O (Valerus Co., Sofia, Bulgaria), which were dissolved in deionized water, transferred into a 50 mL Teflon-lined stainless-steel autoclave, and kept at different temperatures (100, 120, 140, 160 °C) for 2 h. After hydrothermal treatment, the resulting samples were dried at 60 °C for 12 h. The obtained samples are designated as follows: VV0—sample obtained at 100 °C, VV1—at 120 °C, VV2—at 140 °C, VV3—at 160 °C.

### 4.2. Electrode Preparation and Cell Assembly

Negative electrodes made from activated carbon xerogel [49] were prepared by formulating an ink consisting of 75 wt% of this activated carbon (AC), 5 wt% graphite (ABG 1005 EG1), 5 wt% carbon black, and 15 wt% polyvinylidene difluoride (PVDF, Sigma Aldrich, Burlington, MA, USA) in dimethylacetamide (DMAc, Alfa Aesar, Ward Hill, MA, USA). The ink was thoroughly mixed and applied to a glass substrate. The coated film was then dried at 70 °C for 12 h, carefully peeled off from the glass substrate, and subjected to further drying at 120 °C for 1 h and 160 °C for 20 min. Electrodes with an area of 0.785 cm^2^ were punched out from the resulting film for the assembly of activated carbon electrodes used in the electrochemical tests [50].

The synthetized MnO_2_-based powders were used to fabricate self-supporting composite electrodes containing 40–75 wt% MnO_2_, combined with varying proportions of activated carbon xerogel, 5 wt% graphite (ABG 1005 EG1), 5 wt% carbon fibers, and 15 wt% PVDF binder in DMAc solvent. The fabrication procedure was the same as that described for the activated xerogel electrodes.

The electrolyte employed was a perfluorosulfonic acid (PFSA) membrane Aquivion^®^ E87-05S (Solvay, Brussels, Belgium), with an equivalent weight of 870 g/mol and a thickness of 50 µm, commercially available from Solvay, Belgium. Before assembly with two electrodes in a Swagelok cell, the membrane was immersed in a 1 M Na_2_SO_4_ solution for three hours at room temperature to convert it into the Na^+^ form. In addition to serving as an electrolyte, the membrane also functions as a separator in the electrochemical cell.

### 4.3. Physicochemical Characterization Methods

The phase composition of the final materials was checked by X-ray diffraction (XRD) using a Philips X-ray diffractometer PW 1030 (Philips Analytical, Almelo, The Netherlands), with an X-ray source of Cu Kα radiation (λ = 1.5406 Å) and θ-2θ Bragg-Brentano geometry. The scan step size was 0.02°, the collection time was 1 s, and the range was 5–90° 2θ. Diffractogram interpretations were performed using the database PDF 2—2022, ICDD.

The morphology and structure of MnO_2_ were characterized by scanning electron microscopy using Scanning electron microscope EVO10 (Carl Zeiss GmbH, Jena, Germany) and also observed by transmission electron microscopy (TEM) and selected area electron diffraction (SAED) using a JEOL JEM 2100, 80–200 kV (Jeol Ltd., Tokyo, Japan).

N_2_ adsorption–desorption isotherms were performed after overnight degasification of the samples, and the specific surface area was evaluated by applying Brunauer–Emmett–Teller (BET) method, the total pore volume was calculated according to Gurwitsch’s rule, and the pore size distribution was estimated by using the Barrett–Joyner–Halenda method.

### 4.4. Electrochemical Characterization

Electrochemical measurements for each supercapacitor were conducted using cyclic voltammetry (CV) at varying scan rates from 1 to 40 mV/s, within a voltage range of 0 to 1.6 V. The measurements were performed using a PalmSens 4 instrument (CL Houten, The Netherlands). The specific capacitance, Cs, obtained from cyclic voltammetry, was calculated using the formula Cs = [4(I/(dV/dt))/m] (1), where I is the current in amperes, dV/dt is the voltage scan rate in V/s, and m is the mass of the active material in grams. Galvanostatic charge–discharge cycling and long-term durability tests were performed using an Arbin BT-2000 instrument (College Station, TX, USA). The tests were conducted over a voltage range of 0 to 1.6 V, with a current load varying between 60 and 1200 mA/g. Long-term cycling tests were carried out up to 5000 cycles at a current range of 240 mA/g. The specific capacitance, C was calculated using the formula: C = 2I dt/mdV (2), where dt is the discharge time in s and dV is the voltage window in V.

## Figures and Tables

**Table 1:** Unit cell parameters, crystal sizes, and the percentages of the two identified phases in the resulting materials.

Sample	Phases	Quantity [wt %]	Unit Cell Parameters					Crystallite Size		
			a [Å]	b [Å]	c [Å]	β [^o^]	V [Å^3^]	2θ [^o^]	hkl	d [nm]
VV0	K_1.39_Mn_3_O_6_	98.48	14.530 (8)	2.857 (4)	9.457 (7)	128.39	307.79	12.62	−201	11.48
	α-MnO_2_	1.52	9.806 (6)	9.806 (6)	2.874 (8)	90	276.47	37.52	211	3.77
VV1	K_1.39_Mn_3_O_6_	56.20	14.591 (3)	2.871 (2)	9.408 (9)	128.66	307.78	12.62	−201	9.12
	α-MnO_2_	43.80	9.864 (1)	9.864 (1)	2.859 (5)	90	278.23	37.52	211	12.89
VV2	K_1.39_Mn_3_O_6_	54.96	14.544 (9)	2.845 (0)	9.473 (2)	128.54	306.60	12.62	−201	9.4
	α-MnO_2_	45.04	9.861 (4)	9.861 (4)	2.860 (5)	90	278.54	37.52	211	10.53
VV3	K_1.39_Mn_3_O_6_	29.25	14.618 (7)	2.786 (4)	9.477 (5)	129.16	299.32	12.62	−201	7.71
	α-MnO_2_	70.75	9.859 (1)	9.859 (1)	2.863 (5)	90	278.34	37.52	211	15.7

**Table 2:** BET surface area and main texture parameters of the VV0–VV3 samples obtained from N_2_ adsorption–desorption isotherms.

Sample	Synthesis Temperature, [°C]	Surface Area BET [m^2^/g]	Total Pore Volume, [cm^3^/g]	Pore Diameter, [nm]
VV0	100	88	0.17	4
VV1	120	106	0.27	5
VV2	140	113	0.26	4
VV3	160	157	0.28	4

## Data Availability

The original contributions presented in this study are included in the article. Further inquiries can be directed to the corresponding authors.
